# Identification of *Arabidopsis* Meiotic Cyclins Reveals Functional Diversification among Plant Cyclin Genes

**DOI:** 10.1371/journal.pgen.1003508

**Published:** 2013-05-09

**Authors:** Petra Bulankova, Svetlana Akimcheva, Nicole Fellner, Karel Riha

**Affiliations:** 1 Gregor Mendel Institute, Austrian Academy of Sciences, Vienna, Austria; 2 Campus Science Support Facilities, Electron Microscopy Facility, Vienna, Austria; Institut Jean-Pierre Bourgin, UMR1318 INRA-AgroParisTech, France

## Abstract

Meiosis is a modified cell division in which a single S-phase is followed by two rounds of chromosome segregation resulting in the production of haploid gametes. The meiotic mode of chromosome segregation requires extensive remodeling of the basic cell cycle machinery and employment of unique regulatory mechanisms. Cyclin-dependent kinases (CDKs) and cyclins represent an ancient molecular module that drives and regulates cell cycle progression. The cyclin gene family has undergone a massive expansion in angiosperm plants, but only a few cyclins were thoroughly characterized. In this study we performed a systematic immunolocalization screen to identify *Arabidopsis thaliana* A- and B-type cyclins expressed in meiosis. Many of these cyclins exhibit cell-type-specific expression in vegetative tissues and distinct subcellular localization. We found six A-type cyclins and a single B-type cyclin (CYCB3;1) to be expressed in male meiosis. Mutant analysis revealed that these cyclins contribute to distinct meiosis-related processes. While A2 cyclins are important for chromosome segregation, CYCB3;1 prevents ectopic cell wall formation. We further show that cyclin SDS does not contain a D-box and is constitutively expressed throughout meiosis. Analysis of plants carrying cyclin SDS with an introduced D-box motif determined that, in addition to its function in recombination, SDS acts together with CYCB3;1 in suppressing unscheduled cell wall synthesis. Our phenotypic and expression data provide extensive evidence that multiplication of cyclins is in plants accompanied by functional diversification.

## Author Summary

The alteration of haploid and diploid cell generations during the sexual life cycle requires meiosis, a specialized cell division that enables the formation of haploid gametes from diploid cells. Meiosis occurs only once during the life cycle, and the transition from the mitotic to meiotic mode of chromosome partitioning requires extensive remodeling of the cell cycle machinery. The cell cycle progression is driven by cyclin-dependent kinases and associated cyclins that regulate CDK activity and confer substrate specificity. Cyclin gene families have undergone a massive expansion in plants, which has raised the question of whether some of these cyclins evolved specific meiotic functions. We systematically analyzed two cyclin gene families in *Arabidopsis* to identify plant cyclins that are meiotically expressed. We found in total eight cyclins to be expressed in male meiotic cells, and functional characterization revealed their involvement in diverse meiotic processes. Interestingly, none of the cyclins appear to be essential for meiotic progression, indicating that plant meiosis is governed by unorthodox cell cycle regulators.

## Introduction

Meiosis is a prerequisite of sexual reproduction enabling the formation of haploid gametes from diploid precursors. This is achieved through partitioning of a diploid set of chromosomes into four haploid nuclei by two consecutive rounds of chromosome segregation. Key hallmarks of meiosis are homologous chromosomes pairing and recombination during prolonged prophase I, kinetochore mono-orientation and protection of centromeric cohesion in metaphase I, and inhibition of chromosome replication in interkinesis [1], [2]. These mechanisms assure faithful segregation of homologous chromosomes in meiosis I and of sister chromatids in meiosis II. The unique behavior of meiotic chromosomes requires fundamental alteration of the basic cell cycle machinery and installation of specific regulatory mechanisms.

Meiotic division occurs only once during the sexual life cycle and transition from mitosis to meiosis is subject to a complex regulation that varies among organisms depending on their reproductive strategies [3], [4]. In angiosperm plants are gametes produced in flowers that differentiate only late during the life cycle. Plant gametogenesis occurs in the context of rapidly dividing cells that fuel formation of developing floral organs [5], [6]. Meiosis takes place in pollen and megaspore mother cells that differentiate from archesporial cells through several rounds of mitotic divisions. Meiotic programming must be activated before premeiotic S-phase during which a special type of cohesion and factors required induction of meiotic breaks are installed on chromatin [7]. In *Arabidopsis* pollen mother cells (PMCs), meiosis lasts approximately 33 h most of which are taken up by prophase I [8]. Immediately after meiosis, male and female haploid spores undergo two and three rounds of mitotic divisions respectively, in order to produce fully developed gametophytes. Thus, completion of meiosis must be accompanied by inactivation of factors that underlie meiosis-specific processes allowing a rapid switch to a regular mitotic program.

Plants are traditionally used for research of meiosis, especially of meiotic recombination and chromosome segregation [9], [10], [11]. Nevertheless, mechanisms that drive progression into, through, and out of meiosis have not been extensively investigated and are only poorly understood. Several meiotic regulators have been identified in genetic studies. Entry into meiosis depends on the plant specific protein SWITCH/AMEIOTIC1, inactivation of which leads to a failure initiating proper meiosis in *Arabidopsis* and maize [12], [13]. Transition into the second meiotic division requires OSD1, an inhibitor of the anaphase promoting complex (APC) [14], [15]. In addition, *Arabidopsis* possesses a specific regulatory circuit dedicated to meiotic exit and transition to the subsequent mitotic divisions consisting of TDM1 and SMG7 [16], [17], [18]. The *TDM1* and *SMG7* genes act in the same genetic pathway and presumably inhibit cyclin-dependent kinase (CDK) activity at meiotic exit [19].

Activity of CDKs depends on their association with cyclins. Different cyclin-CDK complexes promote progression through distinct cell cycle transitions. In animals, entry into S-phase is under control of D- and E-type cyclins, progression through S-phase depends on A- and E-cyclins and M-phase is orchestrated by A- and B-type cyclins. Budding yeast contains three G1/S cyclins (GLN1-3), and six B-type cyclins (CLB1-6) that are required for passage through S- and M-phases. While cyclins expressed in the same cell cycle phase may be partially redundant, ample experimental evidence supports the view that members of the different cyclin subclasses govern distinct cellular processes, either by virtue of their differential regulation or through contributing to substrate specificity of CDKs [20], [21]. This raises the question whether remodeling of chromosome segregation machinery in meiosis involves utilization of specific cyclins. Data from several model organisms suggests that some cyclins have acquired meiosis specific functions. For example, expression of A1 and B3 cyclins in mouse is restricted to the germ-line and *cyca1* knock-out mice are infertile due to meiotic arrest in diplotene [22]. COSA-1, a cyclin-related protein recently identified in *Caenorhabditis elegans*, appears to be important for conversion of meiotic breaks into cross overs [23]. In budding yeast, five out of six Clb cyclins are expressed in meiosis; however, their expression is restricted to specific meiotic stages by elaborate postranscriptional and posttranslational mechanisms suggesting their functional diversification [24]. Indeed, misexpression of Clb3 interferes with meiosis I and leads to premature sister chromatid segregation. These examples suggest that cyclin specificity is an important factor in meiotic cell cycle reprograming.

Studies in *Arabidopsis* have revealed two cyclins with meiotic functions. *SOLO DANCERS* (*SDS*), a plant cyclin related to A and B-type cyclins, is required for homologous chromosome pairing [25], [26], [27]. TARDY ASYNCHRONOUS MEIOSIS (TAM) is an A-type cyclin (CYCA1;2) whose activity is important for entry into meiotic divisions; *tam* mutants exit meiosis prematurely forming diploid microspores [28], [29]. Nonetheless, TAM does not appear to be an essential component of the core meiotic CDK-cyclin oscillator. It is expressed only during meiosis I and its requirement for entry in meiosis II is alleviated by inactivation of SMG7 or TDM1 [19]. Thus, other cyclins are expected to drive meiotic progression. However, no other meiotic cyclins have been identified in genetic screens. In comparison to yeast and animals, the cyclin gene family has undergone massive expansion in angiosperm plants. Genome annotations revealed 50 cyclins assigned to 10 families in *Arabidopsis*, 49 cyclins were found in rice and 59 cyclins were detected in maize [30], [31], [32], [33]. Plant A- and B-type cyclins are orthologous to their animal counterparts and are functionally linked to S- and M-phases of the mitotic cycle, which predicts their involvement also in meiosis. *Arabidopsis* possesses 10 *CYCA* and 11 *CYCB* genes indicating a functional redundancy among individual cyclins that may preclude identification of meiotic cyclins through mutant analyses. Therefore, in this study we performed systematic immunolocalization and histochemical screen to find meiotically expressed A- and B- type cyclins and to examine their role in meiosis.

## Results

To identify cyclins expressed in meiosis, we created transgenic *Arabidopsis* lines harboring C-terminal fusions of all *Arabidopsis CYCA* and *CYCB* genes to the GUS reporter. The GUS tag serves two purposes: it permits tissue specific localization of cyclin expression by a histochemical GUS assay and allows intracellular detection of cyclin:GUS proteins by immunolocalization with a GUS-specific antibody. The constructs consist of entire cyclin genic sequences including introns and ∼2 kb upstream promoter regions fused to the GUS cDNA at the position of the termination codon. C-terminal tagging is commonly used for animal and yeast cyclins and this strategy was also employed for cyclin localization studies in plants [34], [35]. Furthermore, we were able to fully complement the *tam* phenotype by transforming mutant plants with a *TAM:GUS* construct indicating that the C-terminal GUS tag has no or only a negligible effect on cyclin function (data not shown).

We generated *Arabidopsis* transgenic plants carrying reporter constructs of 10 *CYCB* and of all 10 *CYCA* genes; to monitor expression of CYCB1;1 we used a published reporter line that contains the GUS gene fused to the native promoter along with the first two exons of CYCB1;1 [36]. Approximately 20 independent T1 transgenic plants for each construct were prescreened by histochemical GUS staining of flowers; progeny of three plants with the most representative staining pattern were used for more thorough examination in T2 and T3. Three constructs, CYCA1;1:GUS, CYCB1;5:GUS and CYCB2;5:GUS, did not produce reproducible staining and we did not examine them further. Moreover, RT-PCR failed to detect any transcripts encoding intact CYCB1;5 or CYCB2;5 proteins arguing that these genes likely represent non-functional pseudogenes (data not shown).

Histochemical GUS staining of two week old seedlings containing the remaining reporter constructs confirmed that expression of cyclins is largely confined to tissues with rapidly proliferating cells such as root tips, shoot apical meristems and young emerging leaves (Figures S1, S2, and S3; Table 1). Nevertheless, we observed differences in intensities and staining patterns among individual cyclins and cyclin families. In root tips, expression of B-type cyclins was restricted to the tip-proximal part of meristematic zone (Figure S1). CYCB1:GUS constructs yielded a strong spotty pattern derived from proliferating cells in G2/M phases of the cell cycle. B2 cyclins were less expressed in root tips and a weak signal restricted to several cells was detected only for CYCB2;2 and CYCB2;3. In contrast to CYCB1s and CYCB2s, expression of the CYCB3;1 was more diffuse, although some cell showed stronger staining than others. CYCA1;2 produced pronounced spotty signal that overlapped with expression of CYCBs (Figure S1). Interestingly, expression of the other A-type cyclins detected in roots (A3;1, A3;2, A3;4, A2;3, also weakly in A2;2 and A2;4) was shifted to the transition region between meristematic and elongation zones that is characterized by cells undergoing endoreduplication. With the exception of CYCA2;4, which exhibited stomatal expression, these cyclins were also present in nuclei of developing trichomes (Figure S2). Cyclins A2;1, A3;3, B2;1 and B2;4 were not detected in seedlings.

**Table 1:** Expression of CYC:GUS constructs in reporter lines.

Cyclin	Root	Shoot apex	Inflorescence	Localization in mitotic cells	Meiotic expression
A1;1	−	−	−	-	-
TAM (A1;2)	+++	+++	+++	nucleus	L-P
A2;1	−	−	+	n. a.	L-P
A2;2	+	++	++	nucleus	L
A2;3	+++	++	+++	nucleus	-
A2;4	++	+++	+++	nucleus	-
A3;1	+++	+++	+++	nucleus	-
A3;2	+++	+++	+++	nucleus	L
A3;3	−	−	+	-	L-TII
A3;4	+++	++	+++	nucleus	L
B1;1	+++	+++	+++	chromatin	-
B1;2	+++	+++	+++	chromatin	-
B1;3	+++	+++	+++	chromatin, cytoplasm	-
B1;4	+++	+++	+++	cytoplasm	-
B2;1	−	−	++	cytoplasm	-
B2;2	−	+	++	cytoplasm	-
B2;3	++	+++	+++	cytoplasm	-
B2;4	−	−	+	cytoplasm	-
B3;1	+++	+++	+++	spindle	Z-MI; MII

The data were obtained from assessing at least three independent reporter lines. Relative intensity of GUS staining in root tips, shoot apex and inflorescence is indicated (“−” no staining and, “+” weak staining, “++” intermediated staining, “+++” strong staining). Intracellular localization in mitotic cells corresponds to interkinesis for A-type cylins and metaphase for B-type cyclins. Meiotic stages: L (leptotene), Z (zygotene), P (pachytene), MI (metaphase I), MII (metaphase II), TII (telophase II).

All examined cyclins were expressed in inflorescences, but intensity and staining patterns varied among them (Figure S4, Table 1). All cyclins were to a various degree present in floral buds; while staining of CYCB2;4 was usually limited to 1–2 buds, most cyclins showed a broader expression. CYCA2;1 and A3;3 were detectable in anthers at the meiotic stage and continued to be expressed also in older buds. Some cyclins (B1;1, B1;4, B2;1, B2;3, A2;3, A2;4, A3;1, A3;2, A3;4) were strongly expressed in developing seeds. B1 cyclins also tended to produce intensive staining in anthers of a single older floral bud suggesting a temporal and synchronized expression at a specific stage of pollen development.

We next examined intracellular localization of cyclin:GUS proteins in cells prepared from young floral buds by immunostaining. We have previously showed that TAM:GUS (CYCA1;2) is present in nuclei of a subset of interphase cells that are presumably in G2, and disappears when cells enter mitosis [19]. Remaining A-type cyclins exhibited the same subcellular localization as TAM (Figures S5 and S6, Table 1). A3 cyclins produced a more pronounced nuclear signal than A2 cyclins, which is consistent with the stronger expression of A3 cyclins detected by the histochemical assay.

B-type cyclins exhibited more complex intracellular localization. In general, CYCBs were visible in the cytoplasm of a subpopulation of interphase cells (G2), their level peaked in metaphase and disappeared during anaphase. While cyclins B1;1 and B1;2 showed prominent association with chromatin in prophase and metaphase, cyclin B1;3 localized to both chromatin and cytoplasm (Figure S7). Interestingly, CYCB1;4 exhibited the same localization as B2-type cyclins: these cyclins were depleted from chromatin and appeared to be enriched in the cytoplasmic region that co-localized with the spindle (Figures S7 and S8). CYCB3;1 was specifically associated with the spindle microtubules (Figure S9). The distinct subcellular localization of different classes of B-type cyclins either to chromatin, cytoplasm or to the spindle indicates their functional diversification.

**Figure 1:**
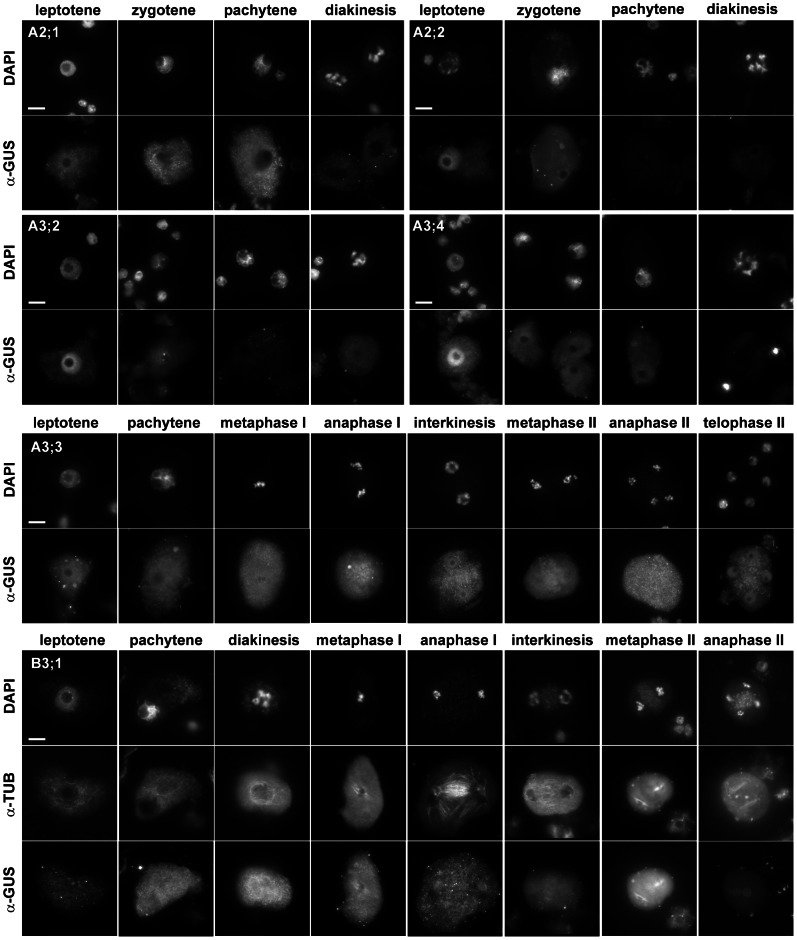
Immunolocalization of CYC:GUS proteins in PMCs. DNA was counterstained with DAPI, microtubules were detected with anti-α-tubulin antibody. Bar = 10 µm.

To determine which cyclins are expressed in meiosis, we looked for the presence of the immunolocalization signal in PMCs. In total, one B- and six A-type cyclins produced specific staining in cells undergoing meiosis (Figure 1, Table 1). We previously showed that TAM is present in the cytoplasm of PMCs from leptotene to pachytene, but diminishes in the later meiotic stages [19]. CYCA2;1:GUS immunostaining is reminiscent of the cyclin TAM. Expression of CYCA2;2, CYCA3;2 and CYCA3;4 was restricted to leptotene where these cyclins preferentially localized to nuclei. CYCA3;3 likely evolved from CYCA3;2, and lost a part of N-terminal domain that includes a D-box. CYCA3;3 was present throughout entire meiosis, but we did not observe any specific signal in somatic cells. The only B-type cyclin expressed in PMCs was CYCB3;1. Its signal was detectable from zygotene to metaphase I, where it localized to the spindle, disappeared in anaphase I, and again reappeared at the spindle in metaphase II (Figure 1).

Identification of meiotically expressed cyclins prompted us to investigate their role in meiotic progression. We were particularly intrigued by CYCA3;3 which appears to be specific for meiosis. In addition, CYCA3;3 also lacks a D-box, the regulatory sequence which is targeted by APC and is essential for cyclin degradation. However, plants carrying a T-DNA insertion disrupting the conserved C-terminus of the cyclin (Figure S10) were fully fertile and we did not detect any obvious meiotic defects. *Arabidopsis* plants carrying mutations in *SMG7* or *TDM1* genes are unable to exit meiosis and we proposed that they are required for inhibiting CDK activity driven by meiosis specific cyclins [19]. Absence of the D-box and constitutive expression throughout meiosis indicated that CYCA3;3 is not degraded via APC and its inhibition at the end of meiosis may rely on a SMG7/TDM1-mediated mechanism. However, we failed to rescue defective meiotic exit in *cyca3;3 smg7* and *cyca3;3 tdm1* double mutants arguing that CYCA3;3 is not target of SMG7/TDM1 regulation. Mutants carrying T-DNA insertions in CYCA3;2 and CYCA3;4 were also fully fertile suggesting no major defects in meiosis. Nonetheless, the *cyca3;4* insertion is placed in 5′UTR and may not impair gene function. Furthermore, CYCA3;2 and CYCA3;4 exhibit very similar expression and intracellular localization and, therefore, they may act redundantly.

**Figure 2:**
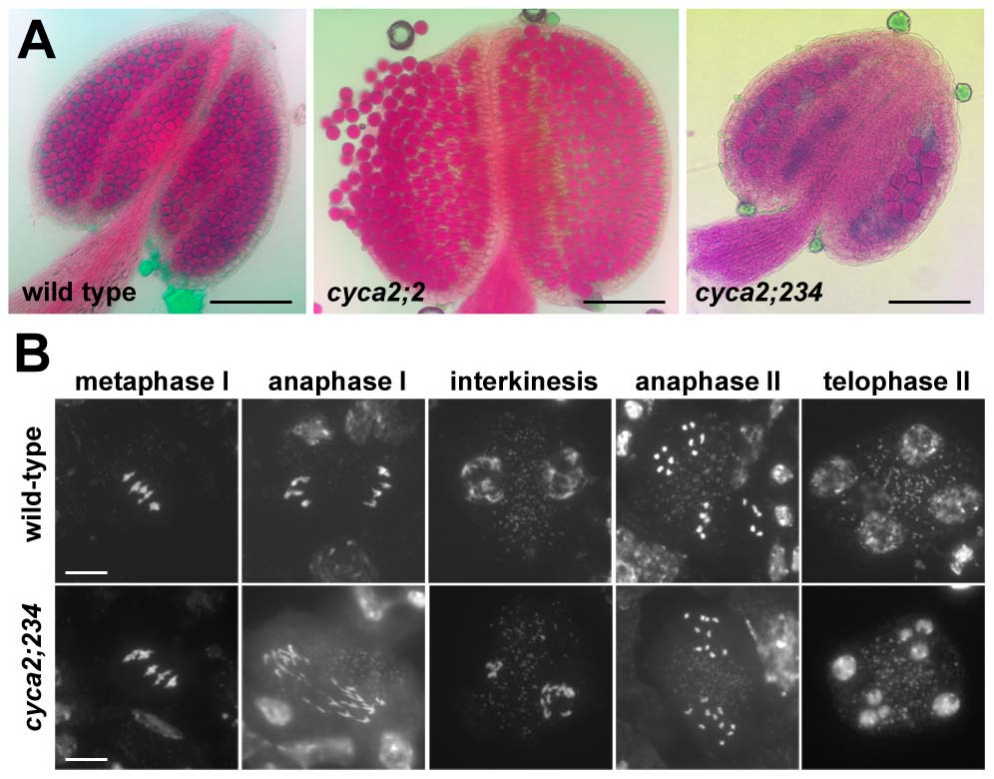
Meiotic defects in *cyca2;234* mutants. (A) Pollen viability in *cyca2;2* and *cyca2;234* mutants determined by Alexander staining. Bars = 100 µm. (B) Meiotic chromosomes stained by DAPI in PMCs from *cyca2;234* mutants. Bar = 10 µm.

Recently, a detailed functional analysis of the *Arabidopsis* A2-type cyclin family was performed using single and combined mutations in all *CYCA2* genes [37]. While single mutants in individual *CYCA2* genes, including *cyca2;1* and *cyca2;2*, were fully fertile, combined mutations in CYCA2;2, CYCA2;3 and CYCA2;4 genes (here referred to as *cyca2;234* mutants) led to stunned growth and reduced seed set (Figure S3 in [37]). To determine whether the underlying cause of reduced fertility stems from aberrant meiosis, we analyzed pollen viability and meiotic spreads from PMCs. Alexander staining showed that *cyca2;234* plants have smaller anthers with less pollen suggesting abortive development of male gametophyte (Figure 2A). Cytogenetic analysis of PMCs did not reveal any obvious aberrations in chromosome pairing during prophase I and *cyca2;234* mutants produced five regular bivalents in metaphase I (Figure 2B). Nevertheless, defects in the chromosome segregation were detected in a portion of meiocytes later during both meiotic divisions. In some meiocytes undergoing anaphase I, we observed segregation of ∼20 partially decondensed chromatids indicating problems with centromeric cohesion and chromatin condensation. We occasionally saw interkinesis nuclei of unequal size suggesting chromosome nondisjunction in meiosis I. We also detected irregularities in anaphase II where separated chromatids appeared to inefficiently migrate to opposite cell poles. Aberrant chromosome behavior was also reflected in a large number of polyads that contained various numbers of unequally sized nuclei. These data demonstrate that the *Arabidopsis* CYCA2 family governs processes important for proper segregation of meiotic chromosomes.

**Figure 3:**
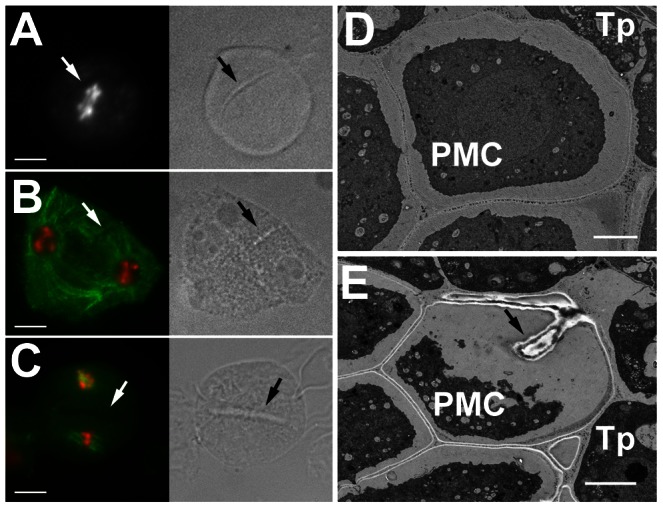
Ectopic cell wall-like structures in *cycb3;1* mutants. (A–C) PMCs from *cycb3;1* plants visualized by epifluorescence (left panel) and bright field (right panel) microscopy. DNA is counterstained with DAPI (white in A, red in B,C), microtubules (green in B,C) were detected with anti-α-tubulin antibody. (A) early anaphase I, (B) interkinesis, (C) metaphase II. Bars = 10 µm. (D,E) Section through anthers with PMCs in late meiosis I visualized by transmission scan electron microscopy. Arrows indicate ectopic cell wall-like structures, c – callose, Tp – tapetum cells. Bars represent 3 µm.

**Figure 4:**
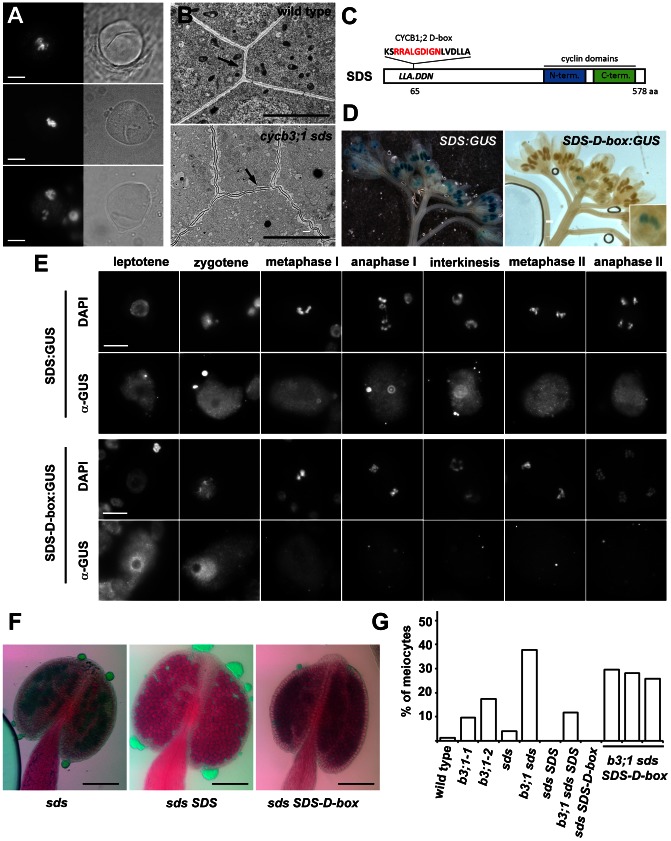
Cyclin SDS contributes to suppressing ectopic cell wall-like structures. (A) PMCs from *cycb3;1-1 sds* mutants visualized by epifluorescence (left panel) and BF (right panel) microscopy. DNA is counterstained with DAPI. Bars = 10 µm. (B) Section through anthers with PMCs in early meiosis I. Arrows point to cell walls separating PMCs. Bars represent 5 µm. (C) Diagram of the SDS protein indicating D-box insertion. (D) Expression of the *SDS:GUS* and *SDS-D-box:GUS* constructs in inflorescence. (E) Immunolocalization of SDS:GUS and SDS-D-box:GUS proteins in PMCs. Bars = 10 µm. (F) Pollen viability determined by Alexander staining in *sds* mutatns complemented with *SDS* or *SDS-D-box* transgenes. Bars = 100 µm. (G) Frequency of meiocytes containing cell-wall like structure. At least 100 meiocytes were counted in each category.

CYCB3;1 is the only B-type cyclin detected in meiosis. This finding was surprising considering a prominent role of these cyclins in mitosis. To elucidate the function of CYCB3;1 in meiosis, we examined two mutant lines carrying T-DNA insertions disrupting the conserved C-terminal cyclin domains (*cycb3;1-1* and *cycb3;1-2* alleles; Figure S10). Plants with these alleles were fully fertile and did not exhibit any obvious growth defects. Nevertheless, we noticed that a fraction of PMCs harbored unusual structures that were apparent by bright field (BF) microscopy and resembled incomplete cell walls formed at ectopic locations (Figure 3, Figure S11). These cell wall-like structures were detected from prophase I through entire meiosis and in some extreme cases appeared to penetrate into chromatin or cut through a spindle (Figure 3B and Figure 4A). Transmission electron microscopy (TEM) revealed unusual cell wall invaginations into the cell interior (Figure 3E) which corroborated bright field microscopy data. These observations indicate that CYCB3;1 activity contributes to spatial and temporal regulation of cell wall formation in PMCs.

Premature meiotic exit accompanied by cell wall formation after meiosis I was reported in *tam* mutants, but we did not detect any irregularities in cell wall location as shown in *cycb3;1* plants suggesting that TAM and CYCB3;1 affect different aspects of cytokinesis. Interestingly, we observed structures resembling parts of ectopically positioned cell-walls by BF microscopy in ∼4% of PMCs in *sds* mutants. Incidence of the affected meiocytes dramatically increased to almost 40% in *cycb3;1-1 sds* double mutants (Figure 4G). Furthermore, examination of anther sections by TEM revealed that cell walls separating meiocytes in *cycb3;1 sds* tend to undulate and are not straight as in wild type plants (Figure 4B). Thus, CYCB3;1 and SDS appear to act redundantly in a pathway controlling cell wall metabolism in PMCs.

SDS was discovered as a cyclin essential for meiotic recombination and chromosome pairing in prophase I [25], [27]. We therefore asked whether SDS function in cell wall formation is coupled to its role in meiotic recombination. Similarly to CYCA3;3, SDS does not contain a D-box and, thus, it may escape APC mediated destruction extending its expression to later meiotic stages. To test this prediction, we fused the GUS reporter to the C-terminus of the SDS gene and generated transgenic plants carrying this construct. Histochemical GUS staining yielded a prominent signal in anthers. Although SDS mRNA was reported to be exclusively expressed in anthers undergoing meiosis [25], we observed strong GUS staining in meiotic anthers as well as in postmeiotic floral buds (Figure 4D). Immunostaining of the SDS:GUS protein showed an uniformly distributed localization throughout entire PMCs (with exception of nucleoli in prophase I) in all stages of meiosis (Figure 4E). To determine whether the broad expression of the SDS is due to protein stability, we inserted a D-box sequence from CYCB1;2 into the N-terminal part of the SDS:GUS construct (Figure 4C). Indeed, presence of the D-box restricted SDS:GUS staining to a single floral bud corresponding to a meiotic stage (Figure 4D). The SDS:GUS immunolocalization signal peaked in leptotene and zygotene and disappeared after pachytene. Interestingly, presence of the D-box led to the enrichment of the SDS:GUS protein in nucleus (Figure 4E).

We next wanted to know whether restricted SDS expression in prophase I affects its meiotic functions. We transformed *sds* and *cycb3;1 sds* mutant plants with SDS genes either with or without the D-box and analyzed fertility and meiosis in complemented plants . Both constructs fully rescued recombination defects and infertility (Figure 4F) demonstrating that expression of SDS in early prophase I is sufficient for its function in meiotic recombination and pairing. In contrast, only the wild-type SDS construct reduced the frequency of PMCs harboring cell-wall like structures in *cycb3;1 sds* plants to the level observed in *cycb3;1* single mutants (Figure 4G); ectopic cell wall formation was still observed in 26–30% of *cycb3;1 sds* meiocytes harboring the SDS-D-box construct. These data argue that SDS combines at least two independent meiotic functions: it is required in early meiosis for proper chromosome pairing, but also acts later in meiosis where it functions together with CYCB3;1 in inhibiting aberrant cell wall formation.

**Figure 5:**
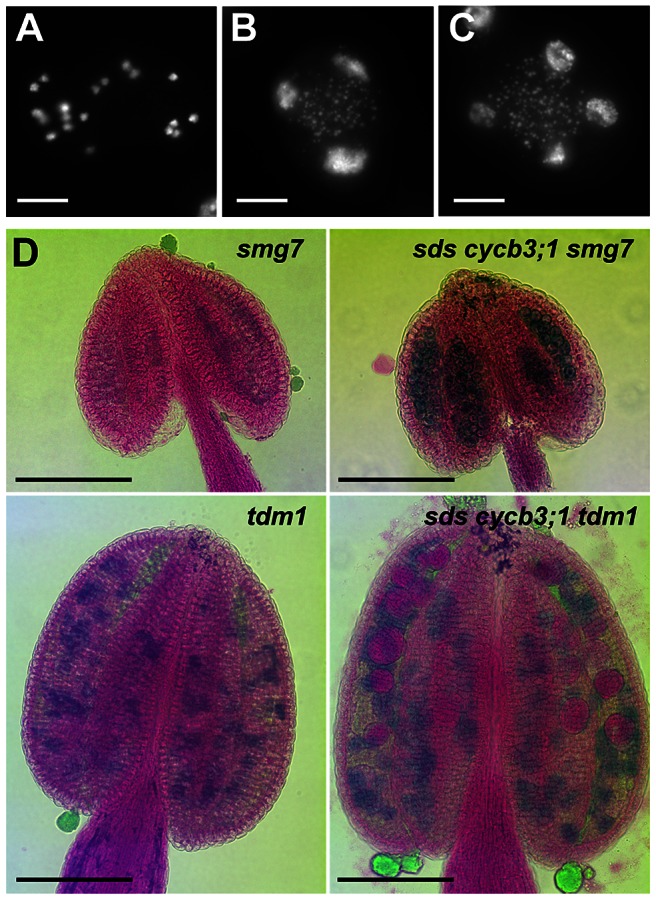
Pollen development in *sds cycb3;1 smg7* and *sds cycb3;1 tdm1* mutants. (A–C) Spreads of PMCs from *sds cycb3;1 smg7* mutants. DNA was stained with DAPI. Besides meiocytes arrested at anaphase II (A), *sds cycb3;1 smg7* mutants contained also PMCs with decondensed nuclei (B,C). Bars = 10 µm. (D) Pollen viability determined by Alexander staining in *sds cycb3;1 smg7* and *sds cycb3;1 tdm1* mutants. Bars = 100 µm.

PMCs in *smg7* mutants arrest in anaphase II due to failed downregulation of CDK activity at meiotic exit and do not form any pollen [18], [19]. Because SDS and CYCB3;1 are expressed in meiosis II, we examined whether their inactivation alleviates the cell cycle arrest by analyzing pollen formation in *sds cycb3;1 smg7* triple mutants. The *sds cycb3;1* mutations did not rescue infertility caused by SMG7 inactivation and we still observed PMCs in aberrant anaphases II stage, which is characterized by condensed separated chromatids (Figure 5A). Nevertheless, we also detected meiocytes in subsequent stages with an irregular number of decondensed nuclei (Figure 5B, 5C) and Alexander staining revealed pollen-like structures in *sds cycb3;1 smg7* anthers (Figure 5D). The *sds cycb3;1* mutations also slightly lessened the severity of *tdm1* phenotypes: while they did not rescue infertility caused by TDM1 deficiency, not all meiocytes in *sds cycb3;1 tdm1* triple mutants aborted and some formed large pollen-like cells apparent after Alexander staining (Figure 5D).These data indicate that SDS and CYCB3;1 at least partially contribute to the CDK activity that hinders meiotic exit in *smg7* and *tdm1* mutants.

## Discussion

Cyclins are essential for CDK activity and their oscillation is critical for ordered and irreversible progression through the cell cycle. Although, in principle, the minimal oscillator sufficient for driving both S and M phases may consist of a single cyclin [38], virtually all eukaryotes possess multiple cyclin genes. This may allow for a flexible control of the cell cycle and its fine-tuning in response to developmental and environmental cues. The cyclin family is particularly enlarged in higher plants with 50 genes annotated within the *Arabidopsis* genome [30], [31]. Temporal and spatial control of cell division plays a pivotal role in plant development as most organs continuously differentiate throughout a plant's life span from rapidly proliferating cells in meristems [39]. Expansion of the cyclin family may therefore reflect complex interactions between cell cycle and developmental decisions. Indeed, several examples in *Arabidopsis* highlight importance of specific cyclins in differentiation of roots, trichomes and stomatal cells [37], [40], [41].

Despite their prominent role in cellular metabolism, most plant cyclins are still uncharacterized and their function is either unknown or only inferred in general terms from animal studies. This is partially due to functional redundancy that complicates genetic analyses [37]. In this study we performed a systematic survey of expression and intracellular localization of all *Arabidopsis* M-type cyclins using C-terminally tagged GUS fusion proteins transcribed from native promoters. We found notable differences in spatial expression patterns between A- and B- type cyclins. B-cyclins are present in meristematic zones with rapidly dividing cells, which is consistent with their role in promoting mitosis, whereas A-cyclins showed more complex localization pattern. Expression of CYCA1;2 is similar to CYCBs inferring its function in mitotic divisions. Cyclins A2 (A2;2, A2;3) and A3 (A3;1, A3;2, A3;4) extend their expression to the root transition zone and developing trichomes that are populated by cells undergoing endocycles. Animal A cyclins promote S phase progression [42] and hence by analogy, the *Arabidopsis* A2 and A3 cyclins may drive the S phase in endoreplicating cells. However, genetic analyses indicate that the CYCA2;3 acts as an inhibitor of endoreplication [41], [43] and this suggestion was recently extended to the whole CYCA2 family [37]. The functional analyses of CYCA3s are limited to ectopic overproduction that results in altered plant morphology and reduced ploidy [44], [45]. Our localization data provide a basis for further experiments aimed on dissecting the role of A-type cyclins in mitotic division and endocycle.

Intracellular localization was reported for some plant A- and B-cyclins [34], [35], [37], [41], [46], but this data was obtained with GFP reporter constructs driven by strong inducible or constitutive promoters, which may result in ectopic expression and mislocalization. Our results with constructs regulated by native promoters show that Arabidopsis A-type cyclins are present in a fraction of interphase nuclei (presumably in the S/G2), but we failed to detect any signal beyond mitotic prophase. This is in agreement with the reported localization of tobacco CYCA3;1 [34]. In contrast, signal from CYCBs is most pronounced in metaphase and disappears in anaphase. Such expression patterns of CYCAs and CYCBs correspond to animal A and B cyclins and supports the view that these cyclin clades are orthologous and diverged before separation of plant and animal lineages [47]. Strikingly, we found distinct subcellular localization among individual CYCB subgroups. CYCB1;1 and CYCB1;2 were enriched on condensing chromatin, whereas CYCB1;4 and all CYCB2s were excluded from chromatin; CYCB3;1 was specifically associated with spindle microtubules. Subcellular targeting of human cyclin B1 is brought about by sequence elements within the cyclin, and the N-terminal was shown to be important for chromatin association [48]. Our data indicate that this feature is also conserved in plants. We show that the CYCB1;1:GUS protein harboring 116 N-terminal amino acids localizes to chromatin and that insertion of a D-box sequence from the N-terminal domain of CYCB1;2 causes nuclear retention of SDS in meiotic prophase. Subcellular localization is an important determinant of substrate specificity of CDK-cyclin complexes [21]. Thus, the different localization of Arabidopsis B-type cyclins indicates their specialization in distinct cellular processes.

**Figure 6:**
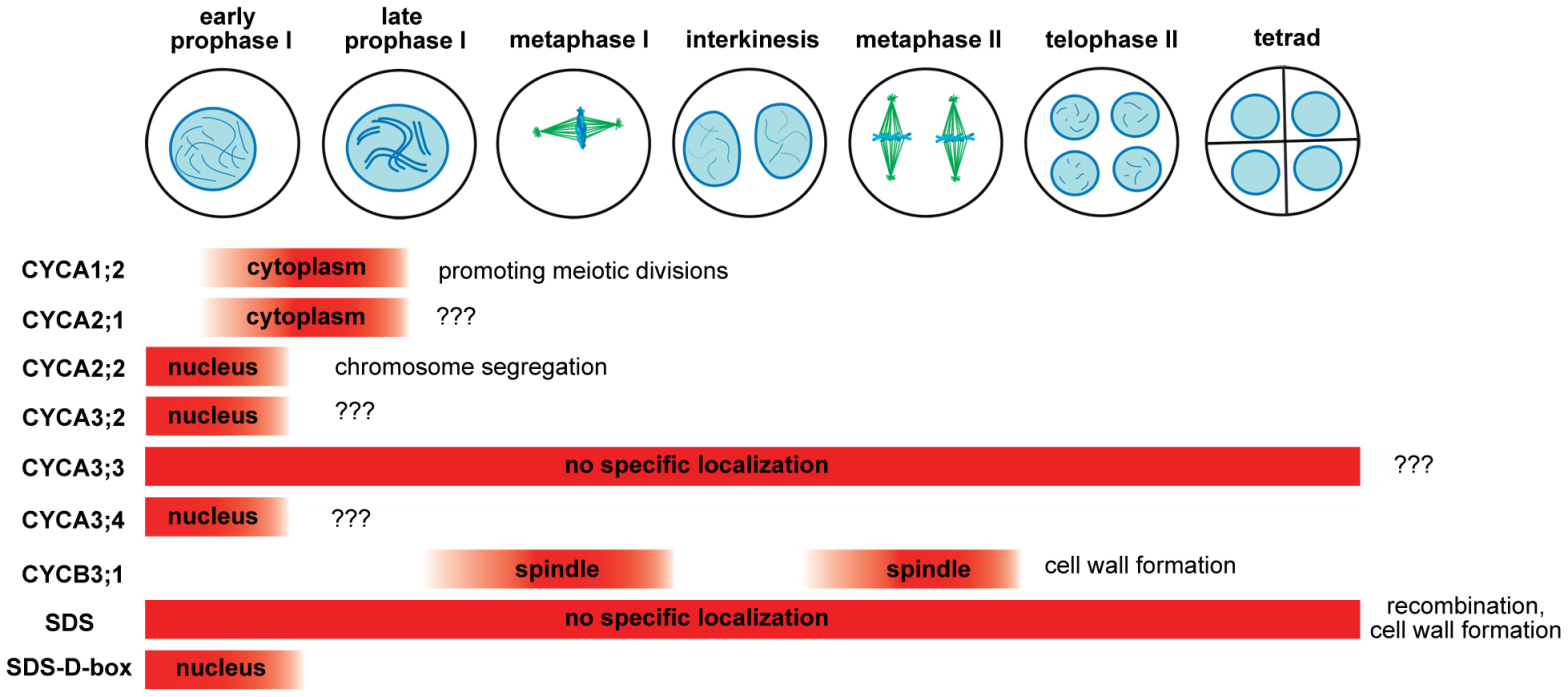
Overview of *Arabidopsis* meiotic cyclins. The diagram depicts expression of cyclins in the course of male meiosis and indicates their role in diverse meiotic processes.

The primary goal of this study was to identify meiotic cyclins. We found in total eight cyclins including SDS to be expressed in meiosis (Figure 6). Because immunodetection has its limitations, we cannot exclude a low meiotic expression (lower than in mitotic cells) for other cyclins as well. Five of the meiotic cyclins (A1;2, A2;1, A2;2, A3;2, A3;4) were restricted to early stages of prophase I. This localization pattern is reminiscent to the situation in mitotic cells where A-type cyclins are present in S/G2 phases and their level declines in prophase (this study, [34]). It is likely that these cyclins are present already in pre-meiotic S-phase where they contribute to processes required for successful segregation of homologous chromosomes in meiosis. This is supported by the observation that *cyca2;234* mutants exhibit chromosome segregation defects that are consistent with aberrant maintenance of sister chromatid cohesion. Although animal and plant A2 cyclins are not orthologous, the mouse A2 cyclin has recently emerged as an important regulator of centromeric cohesion and chromosome segregation in oocytes [49]. Absence of the *Arabidopsis* A-type cyclins, with the exception of CYCA3;3, beyond prophase I may reflect lack of DNA replication in interkinesis.

Two cyclins, A3;3 and SDS, are constitutively present during entire meiosis and their expression seems to last through microsporogenesis (Figure 6). Both cyclins lack the D-box and insertion of the D-box into SDS limits its expression to meiotic prophase I. It is interesting that constitutive presence of these cyclins does not interfere with meiotic progression. For example, mutation of the D-box in TAM that presumably stabilizes the cyclin has detrimental effect on meiosis and aborts meiotic exit [14]. While CYCA3;3 is a relatively recent protein specific to *Arabidopsis sp*., SDS appears to be highly conserved in vascular plants (data not shown). This indicates that SDS possesses very specialized function in meiotic recombination that evolved early in plant evolution. Here we demonstrate a second role of SDS in meiosis that is uncoupled to its function in recombination. We show that SDS together with CYCB3;1 prevents formation of ectopic cell wall like structures during meiosis. Meiocytes in dicot plants undergo unconventional cytokinesis where cell walls form simultaneously after segregation of four haploid nuclei. This process is accompanied by separation of microspores by callose and its subsequent dissolution by callase secreted by tapetum cells. Meiotic cytokinesis is initiated by formation of a number of mini-phragmoplasts that can be nucleated by a relatively small amount of microtubules [50], [51]. It has been reported that injection of an active CDK into stamen hair cells of *Tradescantia* promotes pre-prophase band disassembly [52]. A recent study showed that kinesin NACK1, a key activator of mitotic cytokineisis, is inhibited by CDK-mediate phosphorylation in early mitosis [53]. NACK1 paralog, kinesin NACK2/STUD/TES, is required for the separation of microspores in *Arabidopsis* [54]. It is tempting to speculate that CYCB3;1/SDS dependent CDK activity targets NACK2/STUD/TES pathway in PMCs and prevents ectopic initiation of mini-phragmoplasts and hence, unscheduled cell wall synthesis. This notion is supported by the localization of CYCB3;1 to spindle.

The main incentive of our search for meiotic cyclins was identification of the core cell cycle oscillator. In our previous work we suggested a model in which we predicted existence of the meiotic cyclin/CDK oscillator that is inhibited by the TDM1/SMG7 pathway at the end of meiosis II [19] We anticipated that alleviation of the CDK activity would at least partially rescue *smg7* and *tdm1* associated phenotypes. Surprisingly, our work argues that none of the conventional M-type cyclins are essential component of the core meiotic CDK oscillator. With the exception of CYCA3;3, the A-type cyclins are present only during prophase I and are unlikely to drive progression through meiosis II (Figure 6). Although SDS and CYCA3;3 are expressed throughout meiosis, their combined inactivation neither hinders chromosome segregation in meiosis II, nor substantially rescues *smg7* and *tdm1* associated phenotypes (Figure 5 and data not shown). Mitosis is mainly orchestrated by B-type cyclins, whose levels peak in metaphase. However, the CYCB3;1 is the only B-type cyclin detected in PMCs and its inactivation has relatively a minor effect on meiosis. A marginal role of CYCB3;1 in meiotic progression is further inferred from only a slight alleviation of *smg7* and *tdm1* phenotypes by the *cycb3* mutation. Absence of other B-type cyclins in PMCs is unexpected. Although it is formally possible that these cyclins act redundantly at levels undetectable by our approach but sufficient for meiotic progression, it is more likely that other cyclins or related proteins drive meiotic progression. Examples of such proteins implicated in regulation of important meiotic processes include cyclin-like COSA-1 in *C. elegans*, Dbf4/Cdc7 kinase in budding yeast and RINGO/Speedy in *Xenopus* oocytes [23], [55], [56]. Arabidopsis *RETINOBLASTOMA RELATED* (RBR) protein may also partially contribute to meiotic progression as suggested from the analysis of the *rbr-2* allele that causes defects in meiotic prophase I [57].

Cyclin-CDK complex represents an ancient module that was implemented in the cell cycle control already in the last common eukaryotic ancestor [47]. The number of cyclin and CDK genes has increased over evolution with the growing complexity of multicellular organisms and life strategies. Phylogenetic studies suggest that despite their importance, CDK/cyclin genes are under lower selective constrains against amino acids substitutions allowing for evolutionary flexibility of their functions [58], [59]. The remarkable expansion of the cyclin and CDK gene families in angiosperm plants indicates a high degree of functional diversification. Our systematic analysis of the *Arabidopsis* M-type cyclins provides at least three lines of evidence supporting this prediction. First, we observed cell-type specific differences in expression of many cyclins (e.g. mitotic/meiotic cells, root zones, trichomes). Second, we show localization of cyclins to distinct subcellular compartments, which is most obvious among the B-type cyclins. Finally, mutant analysis revealed that the cyclins expressed in PMCs contribute to different meiotic processes. The SDS cyclin is important for meiotic recombination in prophase I, A2 cyclins appear to orchestrate processes involved in chromosome segregation, TAM promotes transitions to meiosis I and II and CYCB3;1 acts together with SDS in regulating cell wall formation. It is likely that the functional diversification also applies to other cyclins families and it may represent an important evolutionary mechanism used by plants to adapt their growth and life cycle to environmental challenges.

## Materials and Methods

### Plant material growth conditions

Mutants used in this study are listed in Table S1. Mutant plants were genotyped by PCR with primers indicated in Tables S1 and S2. *Arabidopsis thaliana* ecotype Col-0 was used as a control line. In most experiments, plants were grown at 21°C and 60% humidity under long day conditions (16 h light/8 h dark).

### Generation of *CYC:GUS* reporter lines

Cyclin genes were PCR amplified with the iProof Polymerase (BioRad) and primers listed in Table S3. DNA from *Arabidopsis thaliana* ecotype Col-0 was used as a template. *CYCA2;1, CYCA2;2, CYCA2;3, CYCA2;4, CYCA3;2, CYCA3;3 and CYCA3;4* genes were cloned into pENTR/D/TOPO vector (Invitrogen) that contains attL1 and attL2 recombination sites. The fragments then were recombined by the Gateway cloning technology (Invitrogen) in front of the GUS gene in the binary vector pMDC163 [60]. All B-type cyclins, *CYCA1;1*, *CYCA3;1* and *SDS* amplified fragments were cloned in pCR2.1 TOPO vector (Invotrogen) and subsequently subcloned into PstI/XbaI or PstI/BamHI restriction sites in the binary vector pCBK04. Binary vectors were electroporated into *Agrobacterium tumefaciens* GV3101 and *Arabidopsis* Col-0 plants were transformed by the floral dip method. Transformed plants were selected on Grodan supplemented with either 5 mg/L hygromycin (Calbiochem) or 20 mg/L BASTA according to [61]. At least 20 independent T1 transformants were analyzed by histochemical GUS assay [62]; three representative lines were further used for detailed analyses. Seeds of a single representative *CYC:GUS* reporter line for each construct were deposited to the Arabidopsis Stock Centre.

### Histology and cytology

Immunodetection of tubulin and CYC:GUS fusion proteins in PMCs was performed as previously described [19]. Alexander staining for pollen viability was done according to [63]. For detection of GUS fusion proteins by histochemical assay, 10–14 days old seedlings and inflorescences were infiltrated with GUS staining buffer (50 mM Na-phosphate buffer pH 7, 10 mM EDTA pH 8, 0,1% Triton-X-100, 2 mM potassium ferricyanide (K_3_(Fe(CN)_6)_ and 1 mM – 2 mM X-Gluc) and incubated at 37°C for 16 hrs. Samples were washed in ethanol series (20%, 35%, 50%) for 30 min at room temperature and followed by incubation in 70% ethanol for 1–3 hours. Tissues were mounted on slides in 50% glycerol and examined by bright field microscopy.

### Transmission electron microscopy

Analysis of PMCs by TEM was performed as described by [64]. *Arabidopsis* inflorescences were dissected under a stereo microscope and 0.2–0.5 mm long floral buds were transferred into fixative (2.8% (v/v) glutaraldehyde in 0.1 M HEPES buffer (pH 7.2), 0.02% (v/v) Triton-X-100) and incubated for 2 hours at room temperature. Then buds were transferred to fresh fixative and incubated overnight. Samples were wash 3×15 min. in 0,1 M HEPES (pH 7.2), then post-fixed overnight in 1% w/v aqueous OsO4 solution. After post-fixation, the tissue was washed with 3 exchanges of 0.1 M HEPES pH 7.2 for 15 min, dehydrated in graded acetone series with a 10% increment for 15 min in each and embedded in Agar 100 resin. 70 nm sections were cut, post-stained with uranyl acetate and lead citrate and examined with the FEI Morgagni 268D (FEI, Eindhoven, The Netherlands) electron microscope operated at 80 kV. Images were acquired using an 11 megapixel Morada CCD camera (Olympus-SIS, Münster, Germany).

## Supporting Information

Figure S1 Expression of CYC:GUS constructs in root tips. GUS staining was performed in 10–14 days old seedlings.
(TIF)

Figure S2 Expression of CYCA:GUS constructs in seedlings.
(TIF)

Figure S3 Expression of CYCB:GUS constructs in seedlings.
(TIF)

Figure S4 Expression of CYC:GUS constructs in inflorescence. Insets in figures B2;2, B2;4 and B3;1 show enlarged young floral buds.
(TIF)

Figure S5 Immunolocalization of CYCA2:GUS constructs in mitotic cells. CYCA2;1:GUS was not detected in mitotic cells derived from young fluorescence buds. CYC:GUS fusion proteins were detected with α-GUS antibody, DNA was counterstained with DAPI.
(TIF)

Figure S6 Immunolocalization of CYCA3:GUS constructs in mitotic cells. CYCA3;3:GUS was not detected in mitotic cells derived from young fluorescence buds. CYC:GUS fusion proteins were detected with α-GUS antibody, DNA was counterstained with DAPI.
(TIF)

Figure S7 Immunolocalization of CYCB1:GUS constructs in mitotic cells. DNA was counterstained with DAPI, microtubules were detected with anti-α-tubulin antibody and CYC:GUS fusion proteins were detected with α-GUS antibody.
(TIF)

Figure S8 Immunolocalization of CYCB2:GUS constructs in mitotic cells. DNA was counterstained with DAPI, microtubules were detected with anti-α-tubulin antibody and CYC:GUS fusion proteins were detected with α-GUS antibody.
(TIF)

Figure S9 Immunolocalization of CYCB3;1:GUS constructs in mitotic cells. DNA was counterstained with DAPI, microtubules were detected with anti-α-tubulin antibody and CYC:GUS fusion proteins were detected with α-GUS antibody.
(TIF)

Figure S10 Position of T-DNA insertions in the analyzed *CYC* genes.
(TIF)

Figure S11 Wild-type PMC in anaphase I visualized by epifluorescence (left panel) and bright field (right panel) microscopy. DNA is counterstained with DAPI (red), microtubules (green) were detected with anti-α-tubulin antibody.
(TIF)

Table S1 *Arabidopsis* mutants used in the study. T-DNA insertion lines used in this study were obtained from the NASC stock center except for the *cyca2;234* line, which was provided by Dr. Tom Beeckman (VIB, Belgium).
(DOC)

Table S2 Primers used for PCR genotyping.
(DOC)

Table S3 Primers used for PCR amplification of cyclin genes.
(DOC)
