# Supplementary figures and images for: Identification of Arabidopsis Meiotic Cyclins Reveals Functional Diversification among Plant Cyclin Genes
Source: PLoS Genet. 2013 May 9;9(5):e1003508. doi: 10.1371/journal.pgen.1003508 (PMC3649987; doi:10.1371/journal.pgen.1003508)

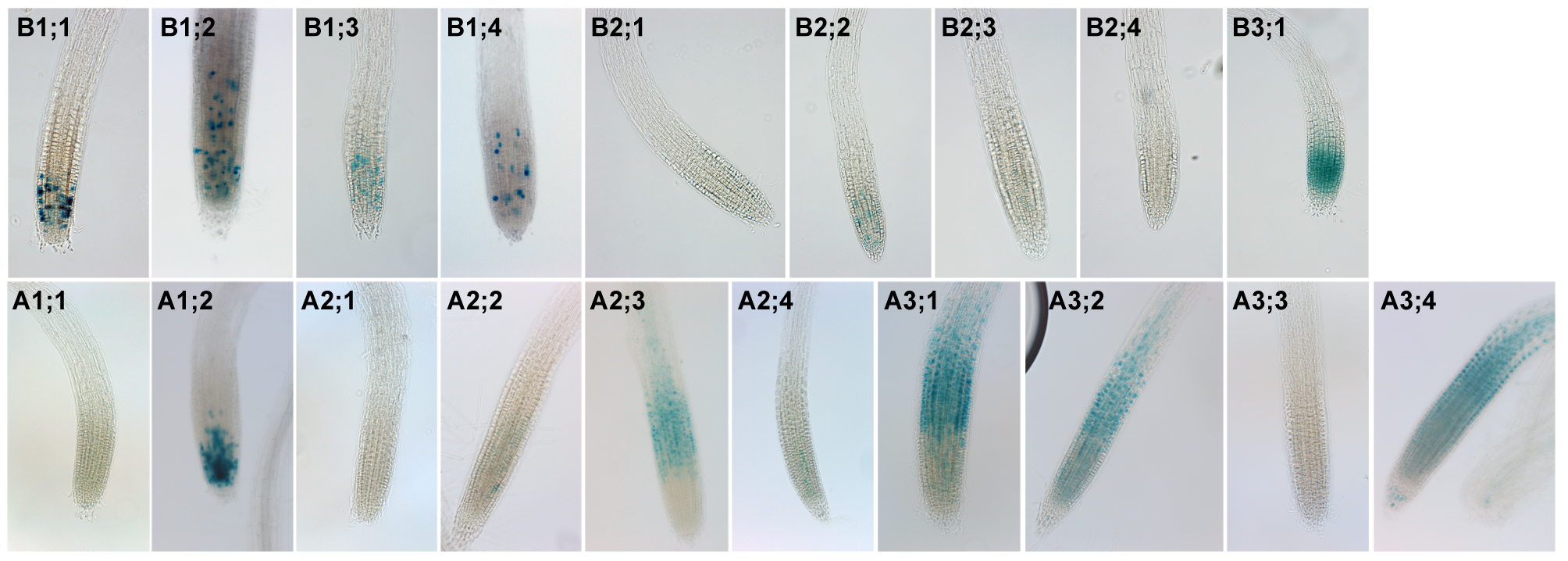

Supplement: Figure S1 — Expression of CYC:GUS constructs in root tips. GUS staining was performed in 10–14 days old seedlings. (TIF) [file pgen.1003508.s001.tif]

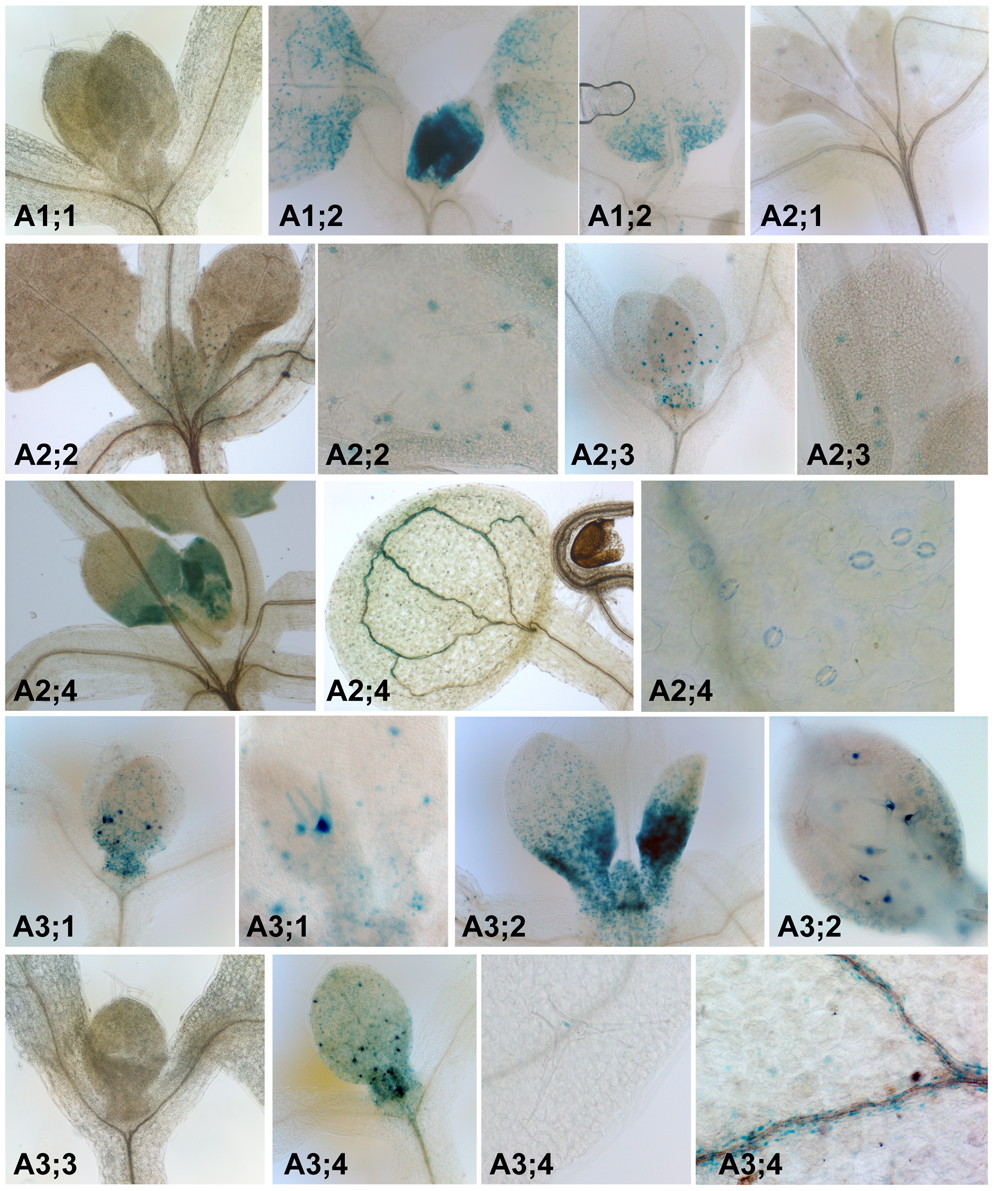

Supplement: Figure S2 — Expression of CYCA:GUS constructs in seedlings. (TIF) [file pgen.1003508.s002.tif]

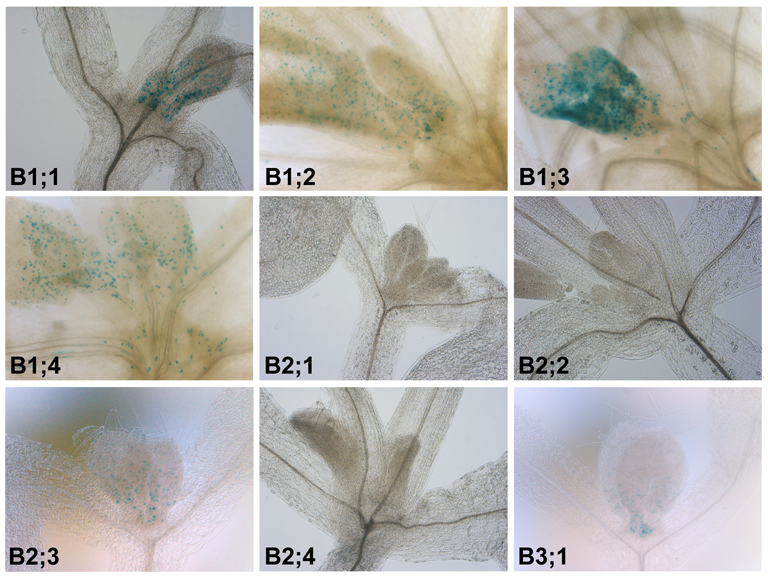

Supplement: Figure S3 — Expression of CYCB:GUS constructs in seedlings. (TIF) [file pgen.1003508.s003.tif]

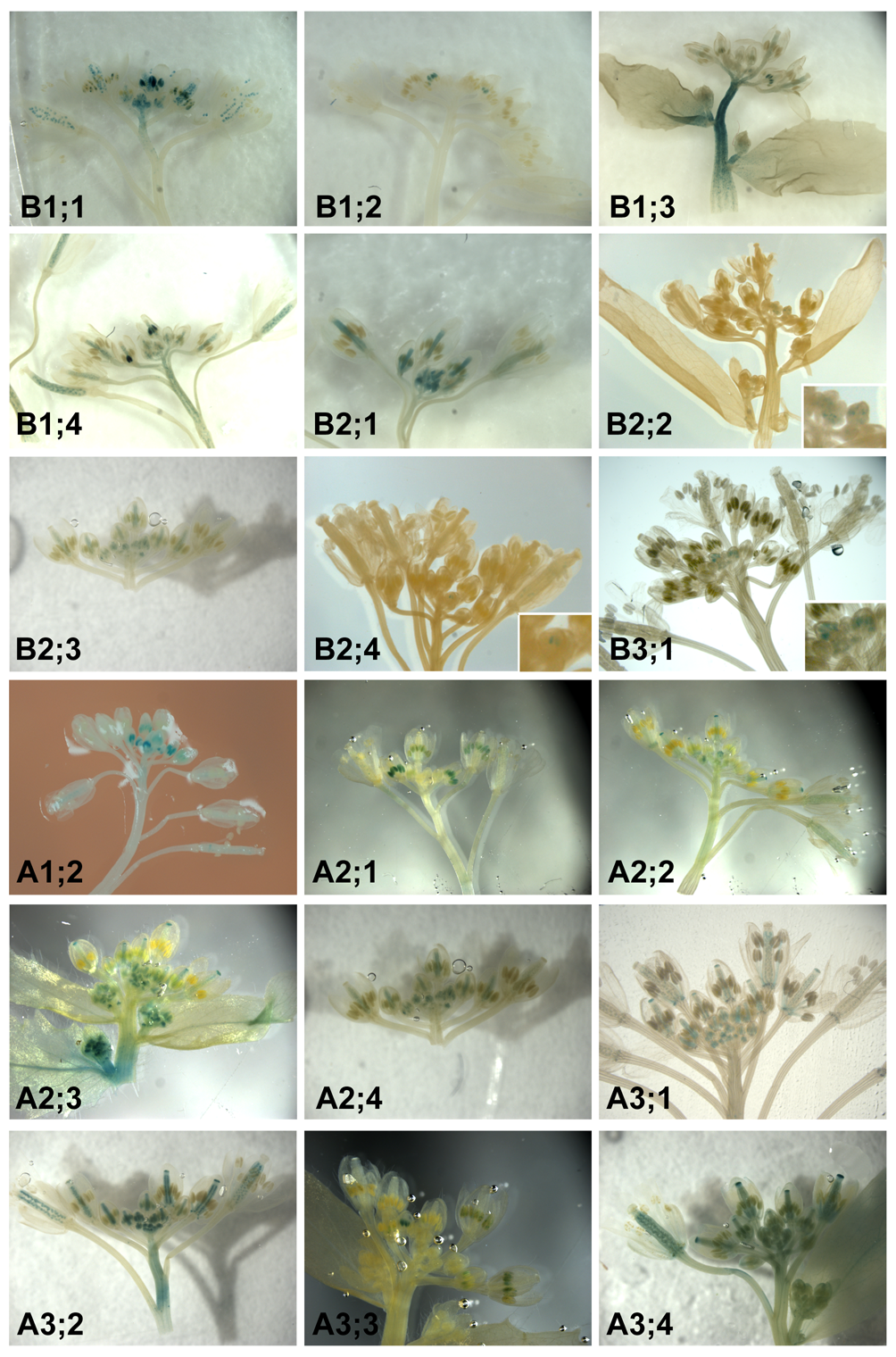

Supplement: Figure S4 — Expression of CYC:GUS constructs in inflorescence. Insets in figures B2;2, B2;4 and B3;1 show enlarged young floral buds. (TIF) [file pgen.1003508.s004.tif]

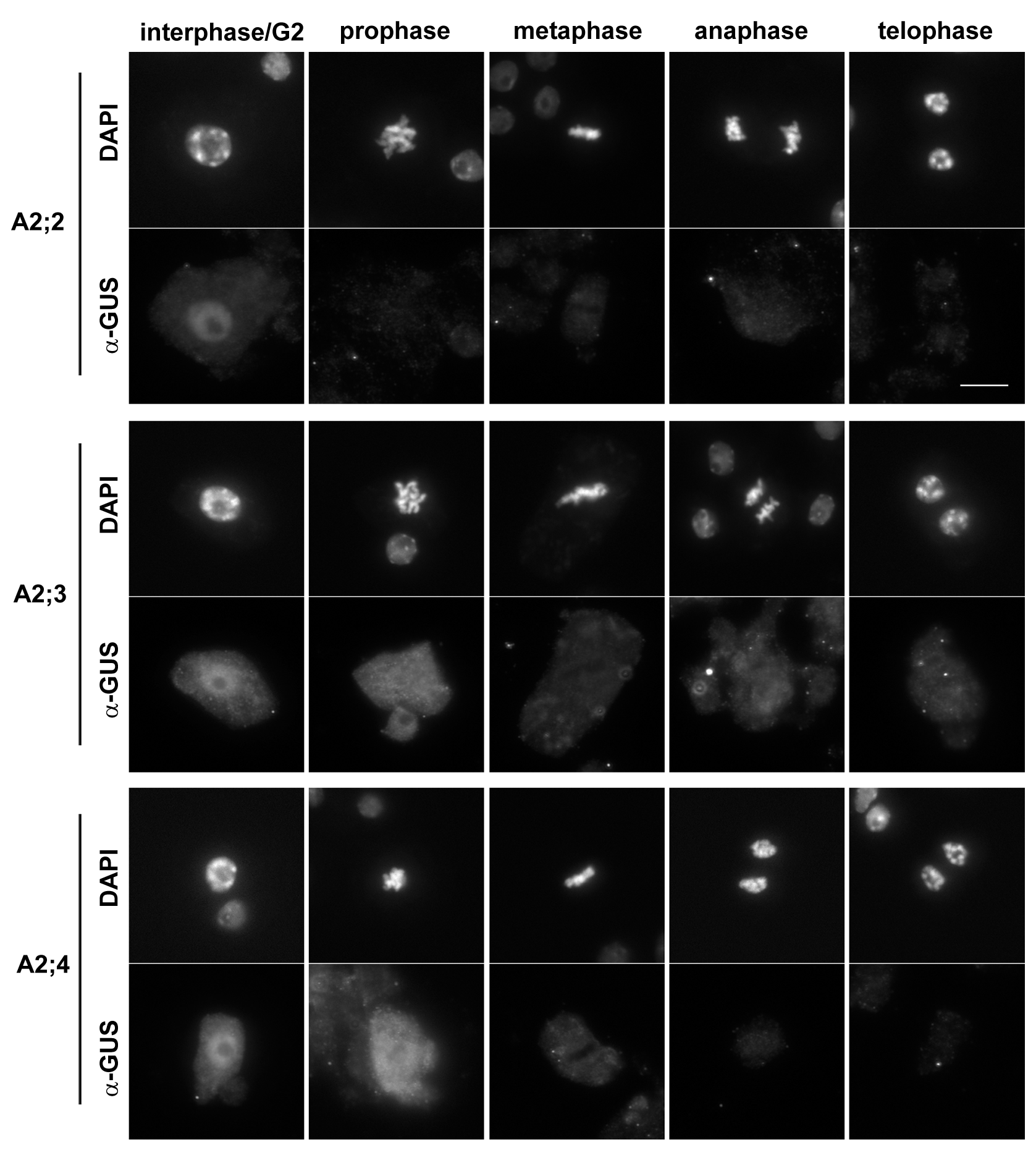

Supplement: Figure S5 — Immunolocalization of CYCA2:GUS constructs in mitotic cells. CYCA2;1:GUS was not detected in mitotic cells derived from young fluorescence buds. CYC:GUS fusion proteins were detected with α-GUS antibody, DNA was counterstained with DAPI. (TIF) [file pgen.1003508.s005.tif]

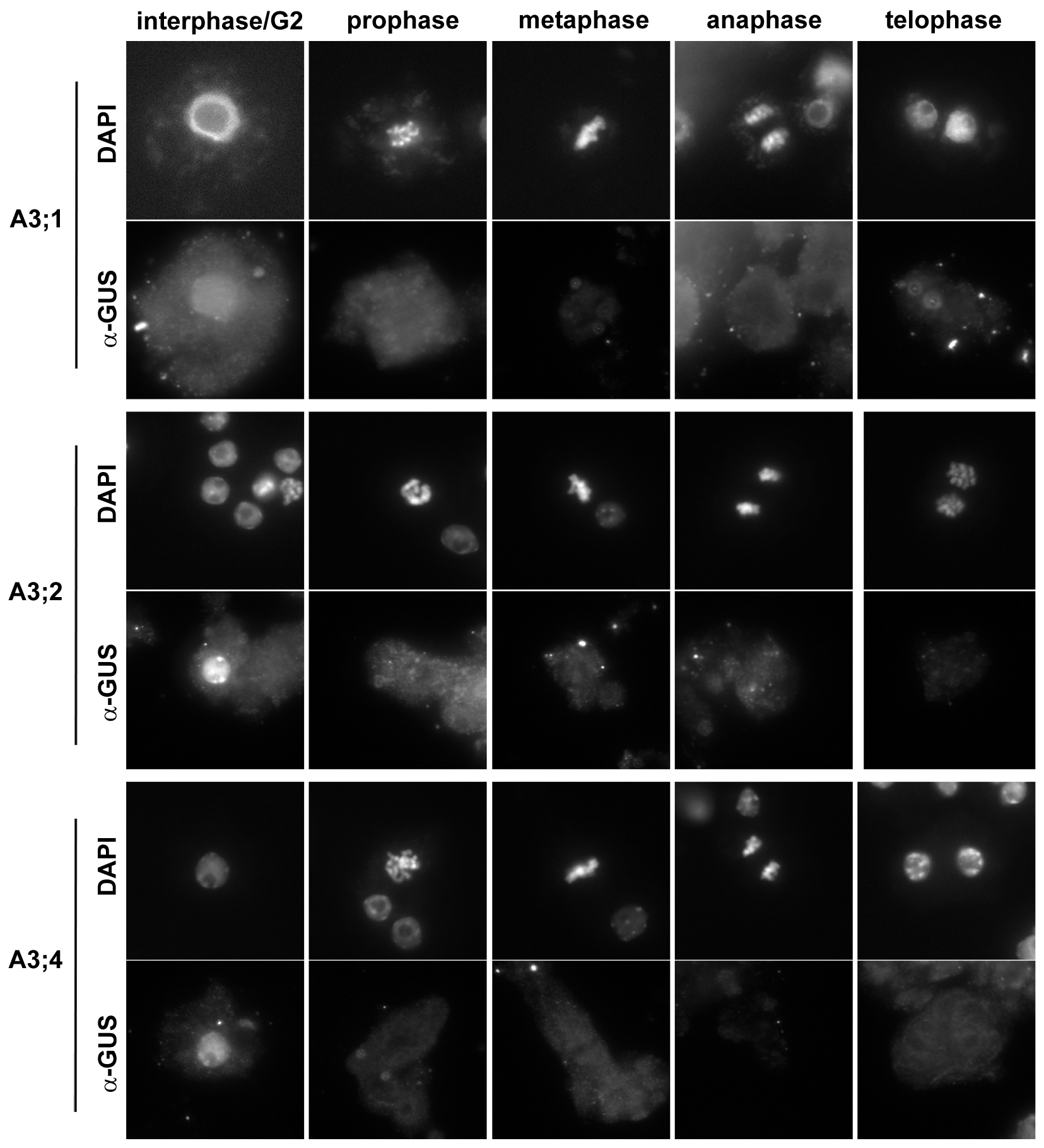

Supplement: Figure S6 — Immunolocalization of CYCA3:GUS constructs in mitotic cells. CYCA3;3:GUS was not detected in mitotic cells derived from young fluorescence buds. CYC:GUS fusion proteins were detected with α-GUS antibody, DNA was counterstained with DAPI. (TIF) [file pgen.1003508.s006.tif]

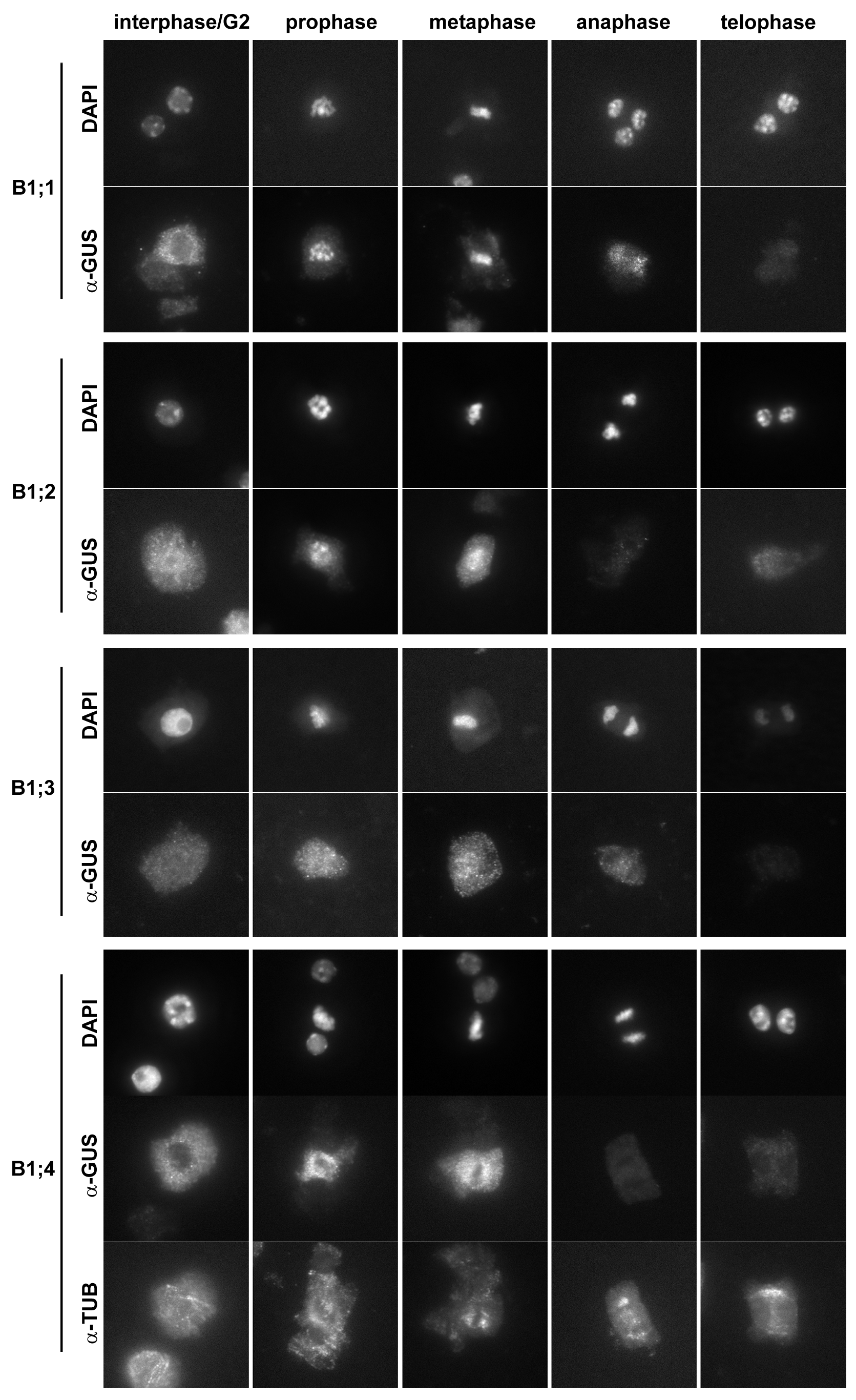

Supplement: Figure S7 — Immunolocalization of CYCB1:GUS constructs in mitotic cells. DNA was counterstained with DAPI, microtubules were detected with anti-α-tubulin antibody and CYC:GUS fusion proteins were detected with α-GUS antibody. (TIF) [file pgen.1003508.s007.tif]

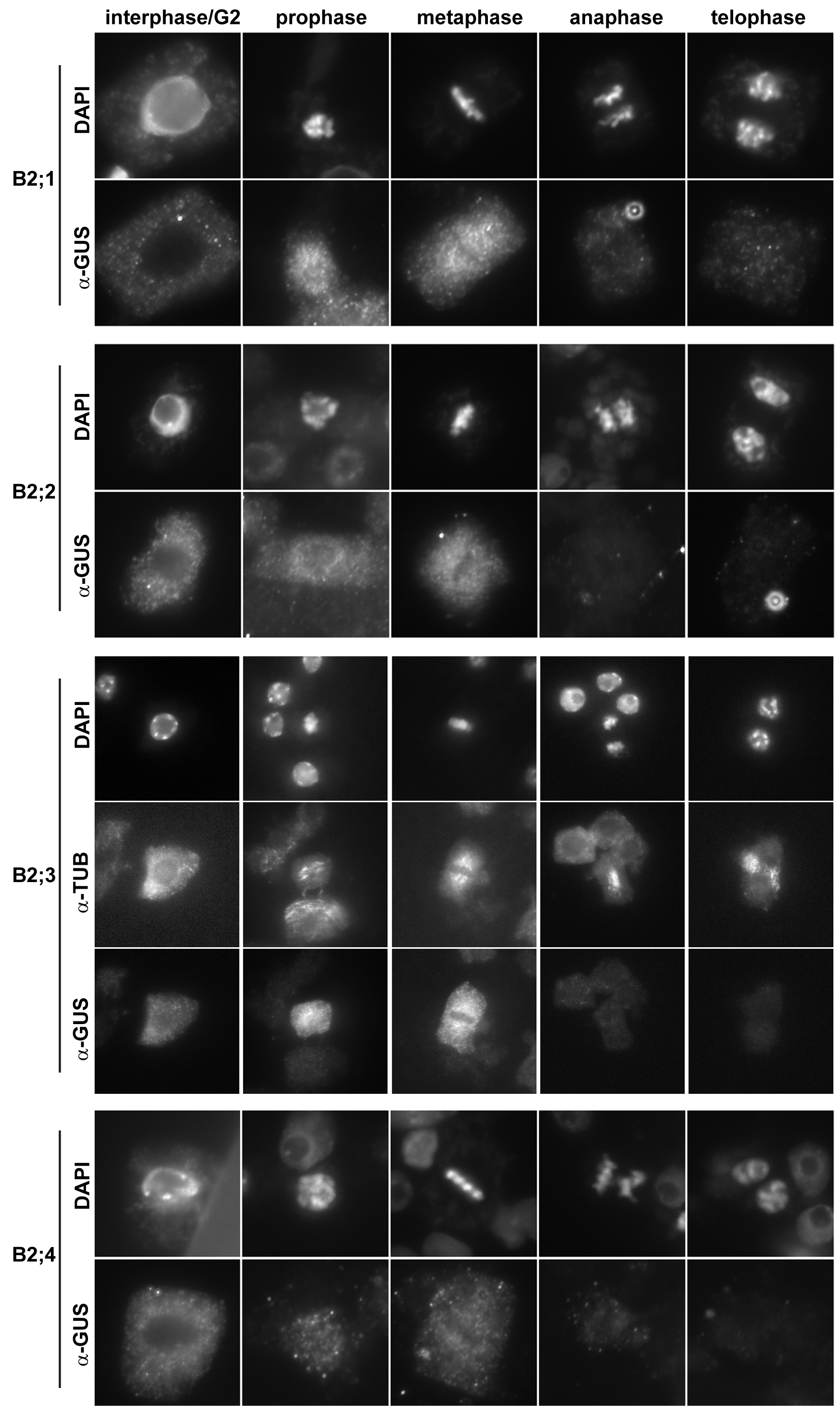

Supplement: Figure S8 — Immunolocalization of CYCB2:GUS constructs in mitotic cells. DNA was counterstained with DAPI, microtubules were detected with anti-α-tubulin antibody and CYC:GUS fusion proteins were detected with α-GUS antibody. (TIF) [file pgen.1003508.s008.tif]

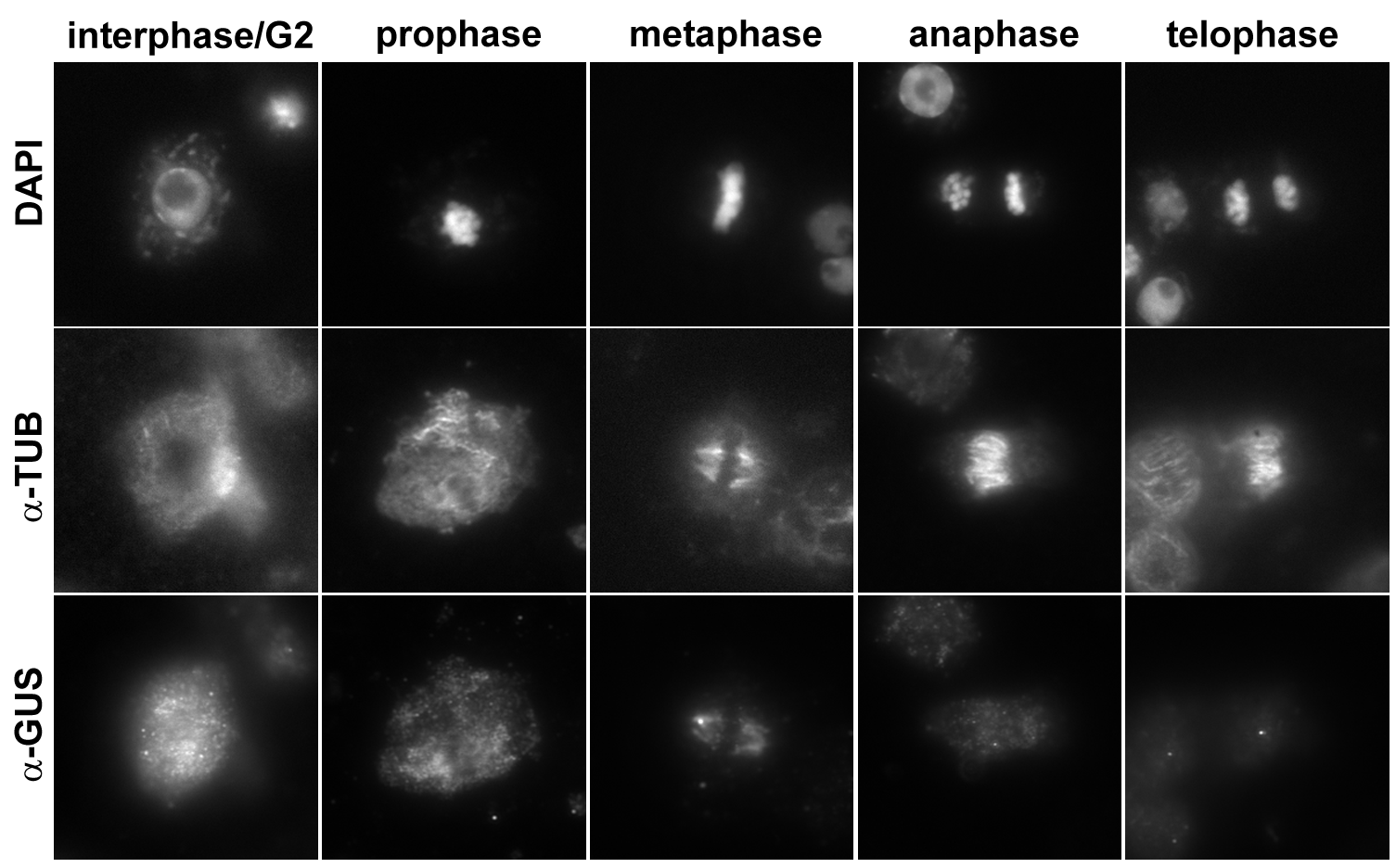

Supplement: Figure S9 — Immunolocalization of CYCB3;1:GUS constructs in mitotic cells. DNA was counterstained with DAPI, microtubules were detected with anti-α-tubulin antibody and CYC:GUS fusion proteins were detected with α-GUS antibody. (TIF) [file pgen.1003508.s009.tif]

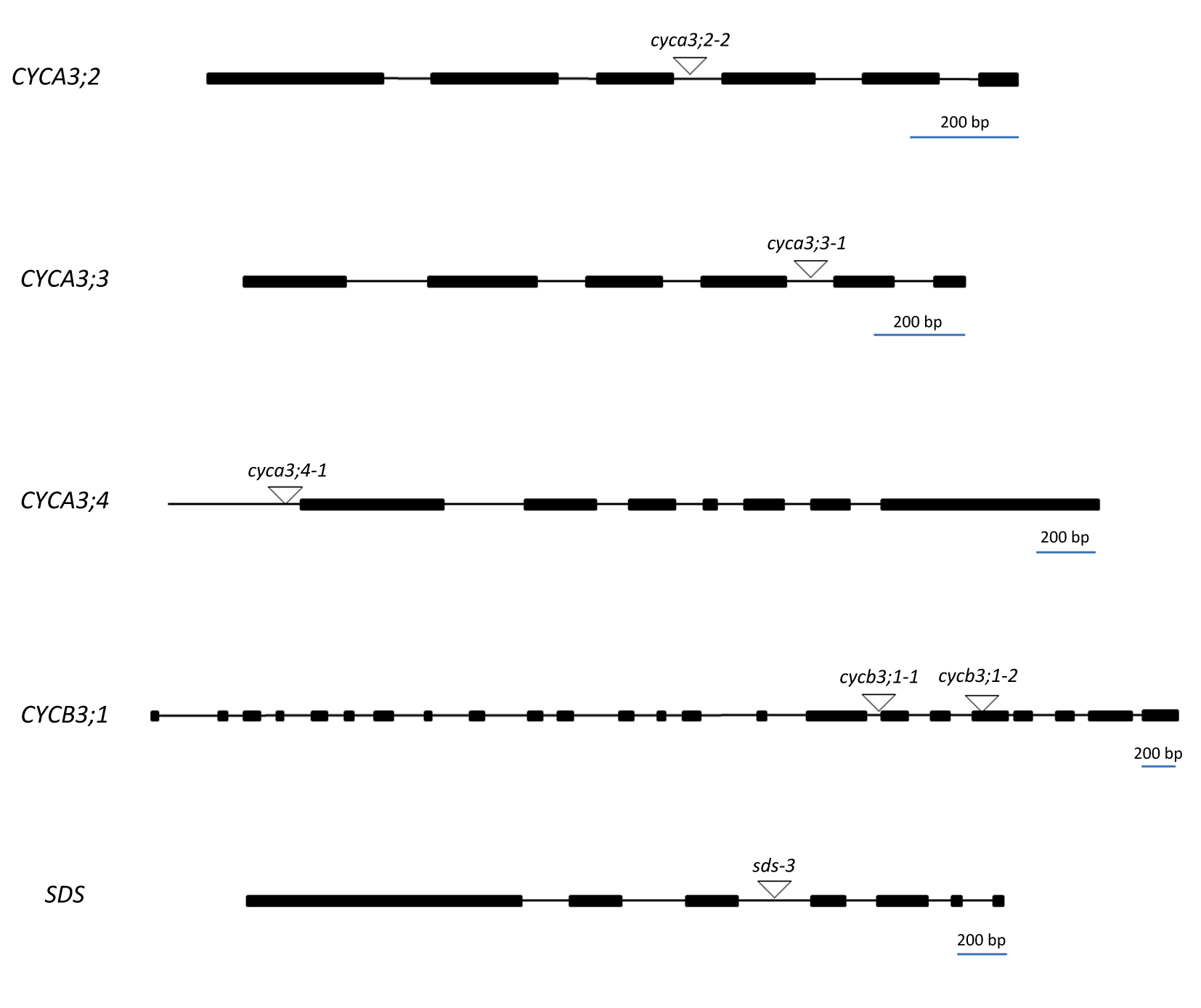

Supplement: Figure S10 — Position of T-DNA insertions in the analyzed CYC genes. (TIF) [file pgen.1003508.s010.tif]

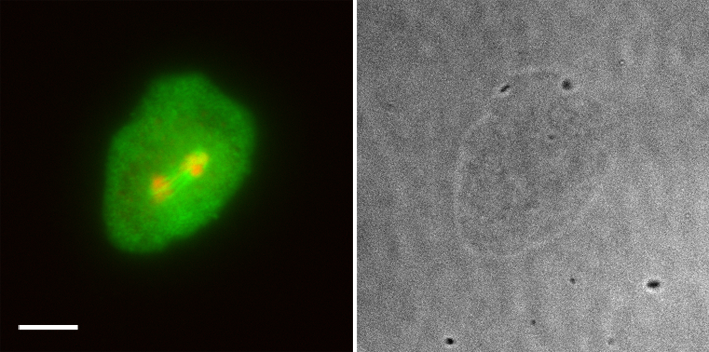

Supplement: Figure S11 — Wild-type PMC in anaphase I visualized by epifluorescence (left panel) and bright field (right panel) microscopy. DNA is counterstained with DAPI (red), microtubules (green) were detected with anti-α-tubulin antibody. (TIF) [file pgen.1003508.s011.tif]
