# Supplementary material for: Identification of Arabidopsis Meiotic Cyclins Reveals Functional Diversification among Plant Cyclin Genes
Source: PLoS Genet. 2013 May 9;9(5):e1003508. doi: 10.1371/journal.pgen.1003508 (PMC3649987; doi:10.1371/journal.pgen.1003508)
Supplement: Table S1 — Arabidopsis mutants used in the study. T-DNA insertion lines used in this study were obtained from the NASC stock center except for the cyca2;234 line, which was provided by Dr. Tom Beeckman (VIB, Belgium). (DOC) [file pgen.1003508.s012.doc]

**Table S1.**

| **Allele** | **AGI code** | **Insertion line** | **Primers for PCR genotyping** | **Reference** |
| --- | --- | --- | --- | --- |
| *cyca2;1-1* | AT5G25380 | SALK_121077C |  |  |
| *cyca2;2-1* | AT5G11300 | GK-120D03 |  | [37] |
| *cyca3;2-2* | AT1g47210 | WiscDsLox246G02 | A32-1, A32-R2, LB-wisc |  |
| *cyca3;3-1* | AT1g47220 | GK-837D11 | A33-R1, A33-5, GABI1 |  |
| *cyca3;4-1* | AT1g47230 | GK-371A12 | A34-3, A34-1, GABI1 |  |
| *cycb3;1-1* | AT1G16330 | WiscDsLox461-464I10 | B31-13, B31-22, LB-wisc |  |
| *cycb3;1-2* | AT1G16330 | GK-955H09 | B31-14, B31-7, GABI1 |  |
| *sds-3* | AT1G14750 | SAIL_129_F09 | SDS-8, SDS-2, SAIL-LB3 |  |
| *smg7-1* | At5g19400 | SALK_073354 |  | [18] |
| *tam-2* | At1g77390 | SAIL_505_C06 |  | [19] |
| *tdm1-1* | At4g20900 | Feldmann line 178 |  | [16,17] |
