# Supplementary material for: Identification of Arabidopsis Meiotic Cyclins Reveals Functional Diversification among Plant Cyclin Genes
Source: PLoS Genet. 2013 May 9;9(5):e1003508. doi: 10.1371/journal.pgen.1003508 (PMC3649987; doi:10.1371/journal.pgen.1003508)
Supplement: Table S2 — Primers used for PCR genotyping. (DOC) [file pgen.1003508.s013.doc]

Table S2.

| **Name** | **Sequence** |
| --- | --- |

| A32-1 | CACTCATCCGATCTCTCAATCCTCT |
| --- | --- |
| A32-R2 | AATGGTAACATCCTCCCAAAAGGTA |
| A33-5 | CATTCAATCAGGCTCTGACATT |
| A33-R1 | AATGTTGACATCTTCAAAAACCGTA |
| A34-1 | CTCCATTTCACGGAGATAAGCACA |
| A34-3 | AGCATTCATTGATTGTTATT |
| B31-13 | CGGTCTAATAAGTCCATTGTCAGGTA |
| B31-22 | TTCTTTTATCGTTTGTGGGCAGCAA |
| B31-7 | TGCATTCAAACGGAACTTTAACTGTT |
| B31-14 | GTGGATGACATATATCAGTTCTATT |
| SDS-2 | CTGCCCAAGCAACCAGTCCAGACT |
| SDS-8 | TCCAGGATGGACAATACTGGTCTCAT |
| Lbc1 | TGGACCGCTTGCTGCAACTCT |
| LB-wisc | AACGTCCGCAATGTGTTATTAAGTTGTC |
| GABI-1 | GATGTTAGGCCAGGACTTTGAA |
| SAIL-LB3 | TAGCATCTGAATTTCATAACCAATCTCGATACAC |
