# Supplementary material for: Identification of Arabidopsis Meiotic Cyclins Reveals Functional Diversification among Plant Cyclin Genes
Source: PLoS Genet. 2013 May 9;9(5):e1003508. doi: 10.1371/journal.pgen.1003508 (PMC3649987; doi:10.1371/journal.pgen.1003508)
Supplement: Table S3 — Primers used for PCR amplification of cyclin genes. (DOC) [file pgen.1003508.s014.doc]

**Table S3.**

| **Name** | **Sequence** | **PCR product (bp)** |
| --- | --- | --- |
| CycA2,1-F1 | CACCGGACTATGATCACATTATGAGAA | 5509 |
| CycA2,1-R1 | TCTTGAGAAGAGTGTGTTCACACGTT |
| CycA2,2-F1 | CACCTTCTGCCGCCGTTTATTAGAA | 4860 |
| CycA2,2-R1 | TCTTGAGAATAGTGATGTGACTCGTT |
| CycA2,3-F1 | CACCCATTGCACTGTAGATCATAT | 5978 |
| CycA2,3-R1 | GAATAGCGTGTCAAGTAGCTTTGGA |
| CycA2,4-F1 | CACCCACTGGCTAGTATGCAAGAA | 6141 |
| CycA2,4-R1 | GGAGATGAATAGCTTGTCCGGTA |
| CycA3,2-F2 | CACCATCTTAAACTTACGTT | 3641 |
| CycA3,2-R3 | AACATCCTCCCAAAAGGTA |
| CycA3,3-F1 | CACCagcattgagcctactactgta | 2331 |
| CycA3,3-R1 | aatggtaacaacatcctcccaaaaggta |
| CycA3,4-F1 | CACCgaaccaatctttcatttcccaa | 3388 |
| CycA3,4-R1 | cgccattcctctaatggtaatatctt |
| A11-PstI-F4 | CTGCAGATCCAAGTTTCTCAATGAATT | 5124 |
| A11-BamHI-R1 | GGATCCGCTGTTGTTGAAGAACTCTTGT |
| A3,1-PstI-F1 | CTGCAGGTGGTGGGTGGGTGATAACTGAT | 5011 |
| A3,1-BamHI-R1 | GGATCCAATGTTGACATCTTCAAAAAC |
| B12For(SbfI) | CCTGCAGGTGTAAGAATCAATCACTAAAGTTTTCA | 2827 |
| B12Rev(NheI) | GCTAGCAGAAGAAACAGGCTTCTTCCAAT |
| B13For(SbfI) | CCTGCAGGTGTTTAGGAAAAAGTTTAATTCAT | 3130 |
| B13Rev(NheI) | GCTAGCTGGAGCAGATGACATAAGAGA |
| B14For(SbfI) | CCTGCAGGAAACCCGTCAATTCTACGATT | 4420 |
| B14Rev(NheI) | GCTAGCTGCACAAGAAACAGAGAAGT |
| B15For(SbfI) | CCTGCAGGTCTCTCCTTTTCCCATCTCTTC | 7365 |
| B15Rev(NheI) | GCTAGCGATCTTCTTTGCAGCTAAACACT |
| B21For(SbfI) | CCTGCAGGATGTCGGGTCAAAAGTGCCTAA | 4675 |
| B21Rev(NheI) | GCTAGCAGAATGATGAGACTCAGACACTA |
| B22For(SbfI) | CCTGCAGGTCGCACTTTAGTCTAGGGATTG | 3548 |
| B22Rev(NheI) | GCTAGCGTGAGAATCTGACACAAGAAAGT |
| B23For(SbfI) | CCTGCAGGTCACTTGTAGGATTACTCAT | 4809 |
| B23Rev(NheI) | GCTAGCAATCAGAAACCCAGCTGGTTCAGTT |
| B24For(SbfI) | CCTGCAGGTCTTCACCGGAATTGTTTTTGA | 3265 |
| B24Rev(NheI) | GCTAGCCAGCAGAAGAAACCCAGCT |
| B25For(SbfI) | CCTGCAGGTCGATCTCTCACGTTCTCTCTAT | 1931 |
| B25Rev(NheI) | GCTAGCcttattatagtTTTTTCCTCCCT |
| B31-F2-SbfI | CCTGCAGGCTTGTCGGAGCAATAAGCATTA | 5598 |
| B31Rev(NheI) | GCTAGCGAGAGGGAGTTTATCTAAGGGC |
| SDS-SbfI-F | CCTGCAGGTAACATGAACAACTGTT | 4680 |
| SDS-Xba-R | TCTAGACTGCCCAAGCAACCAGTCCA |
